# Coexistence of Lichen Sclerosus Et Atrophicus and Morphea in the Same Lesion: A Case Report

**DOI:** 10.7759/cureus.43062

**Published:** 2023-08-07

**Authors:** Styliani Siskou, Ourania Drongoula, Julia Grammatikopoulou, Paraskevi Nivatsi, Despina Noukari, Aikaterini Kokarida, Charikleia Lydia Chrysoglou, Sofia-Ifigeneia Chrysoglou, Periklis Vounotrypidis, Georgios Demirtzoglou, Maria Goula

**Affiliations:** 1 Department of Dermatology, State Hospital for Venereal and Skin Diseases, Thessaloniki, GRC; 2 Department of Plastic and Reconstructive Surgery, KAT Attica General Hospital, Athens, GRC; 3 Laboratory of Medical Biology and Genetics, Faculty of Medicine, Aristotle University School of Health Sciences, Thessaloniki, GRC; 4 Department of Rheumatology, 424 General Military Hospital, Thessaloniki, GRC; 5 Department of Rheumatology, Attikon General University Hospital of Athens, Athens, GRC

**Keywords:** punch biopsy, atypical rash, inflammatory dermatosis, morphea, lichen sclerosus et atrophicus

## Abstract

Lichen sclerosus et atrophicus (LSA) is an inflammatory dermatosis of unknown etiology, usually affecting the genital region, with extragenital involvement being uncommon. The coexistence of LSA and morphea in the same lesion is rare. The present study aims to demonstrate that LSA and morphea might share similar pathologic processes. We present a case of a 53-year-old female patient with extragenital lesions with clinical appearance and histopathological features of both LSA and morphea. Finally, the two diseases might lie on the same disease spectrum.

## Introduction

Lichen sclerosus et atrophicus (LSA) is a chronic inflammatory skin disorder. Its most frequent presentation is in the genital regions of the body [1]. Lichen sclerosus et atrophicus affects mainly premenarchal girls and postmenopausal women [1]. Extragenital LSA is considered to be a rare disease subtype mainly found in females [1]. Its incidence is 0.1%-0.3% but is considered to be underestimated [1]. Extragenital LSA affects approximately 15% of LSA patients. It is estimated that only 6% of LSA patients present with extragenital lesions [1]. Its etiology is not fully understood, but genetic predisposition, epigenetic changes, trauma, certain drugs (beta-blockers and immune checkpoint inhibitors), and hormone changes may play an important role [1]. Also, HLA-DQ7 and HLA-DR12 are involved in LSA’s susceptibility [1]. It is also characterized by global hypomethylation in the dermis and altered isocitrate dehydrogenase activity [1]. Lichen sclerosus et atrophicus is probably a type 1 T helper (Th1) response, and it involves alterations in miR-155 expression of activated immune cells, resulting in loss of immune tolerance by regulatory T cells (Tregs), CD4+ T cell autoreactivity, and sclerotic tissue formation [1]. Autoantibodies against extracellular matrix protein 1 (EMC1) and molecules of the basement membrane zone (BMZ) are found, but their role in disease pathogenesis is not fully understood [1]. Oxidative deoxyribonucleic acid (DNA) damage in LSA results in inflammation maintenance too. Extragenital LSA typically presents with porcelain white atrophic plaques that may be hyperpigmented or hypopigmented [1]. It mainly affects the neck, shoulders, trunk, proximal extremities, flexor surfaces of the wrists, and sites of trauma or pressure [1]. The diagnosis is usually clinical. The gold-standard therapy for this is the application of topical corticosteroids and, alternatively, topical calcineurin inhibitors [1]. The application of systemic corticosteroids and/or methotrexate is indicated for widespread or refractory disease [1].

Morphea, or localized scleroderma, is a rare inflammatory connective tissue disorder characterized by a relapsing and remitting course. Its incidence is estimated to be four to 27 new cases per million [2]. Two incidence peaks are described in morphea. The first one is observed in childhood (two-14 years) and the second one in the fifth decade of life, affecting mostly women [2]. It manifests as inflammatory patches of thickened skin almost everywhere on the body, and depending on the extent and depth of fibrosis, it is classified into five main types (limited, generalized, linear, deep, and mixed) [2]. Each type has several subtypes, like plaque-type morphea and guttate morphea. The pathogenesis of the disease is not fully understood [2]. Genetic and epigenetic factors, infections, skin trauma, autoimmune dysregulation, and vascular dysfunction contribute to morphea pathogenesis [2]. Three different phases of the disease are described. These are the early inflammatory phase, the fibrotic phase, and the atrophic phase [2]. Genetic predisposition is well characterized in morphea, with strong associations with DRB1*04:04 and HLA-B*37 [2]. In the early stages of the disease, Th1/Th17 proinflammatory cytokines play an important role in disease progression and endothelial dysfunction [2]. During disease progression, a swift Th2 immune response is observed, causing skin fibrosis [2]. The diagnosis of morphea can be based on clinical findings, but histopathologic and imaging studies can be useful for diagnosis confirmation [2]. A lot of autoantibodies have been observed in morphea, including antinuclear (ANA), anti-histone (AHA), and anti-single-stranded DNA (ssDNA) antibodies. Disease activity measurement using a localized scleroderma cutaneous assessment tool is crucial for treatment initiation [2]. Morphea treatment is still a clinical challenge. Disease extension, type, severity, and extracutaneous involvement define the management of the disease. General non-pharmacological measurements, topical corticosteroids and/or tacrolimus, or systemic therapy with methotrexate and/or systemic corticosteroids are the main treatment options [2].

Although the concomitant occurrence of LSA and morphea are well established in the literature [3], the existence of both diseases in the same lesion is rare [4]. Herein, we present a case report of a patient with histopathological features of both LSA and morphea at the same lesion.

## Case presentation

We present the case of a 53-year-old female patient with a one-year history of multiple, asymptomatic, gradually enlarging sclerotic plaques of the trunk and extremities. The patient had no comorbidities. She was not receiving any drugs, and her family history was unremarkable.

**Figure 1:**
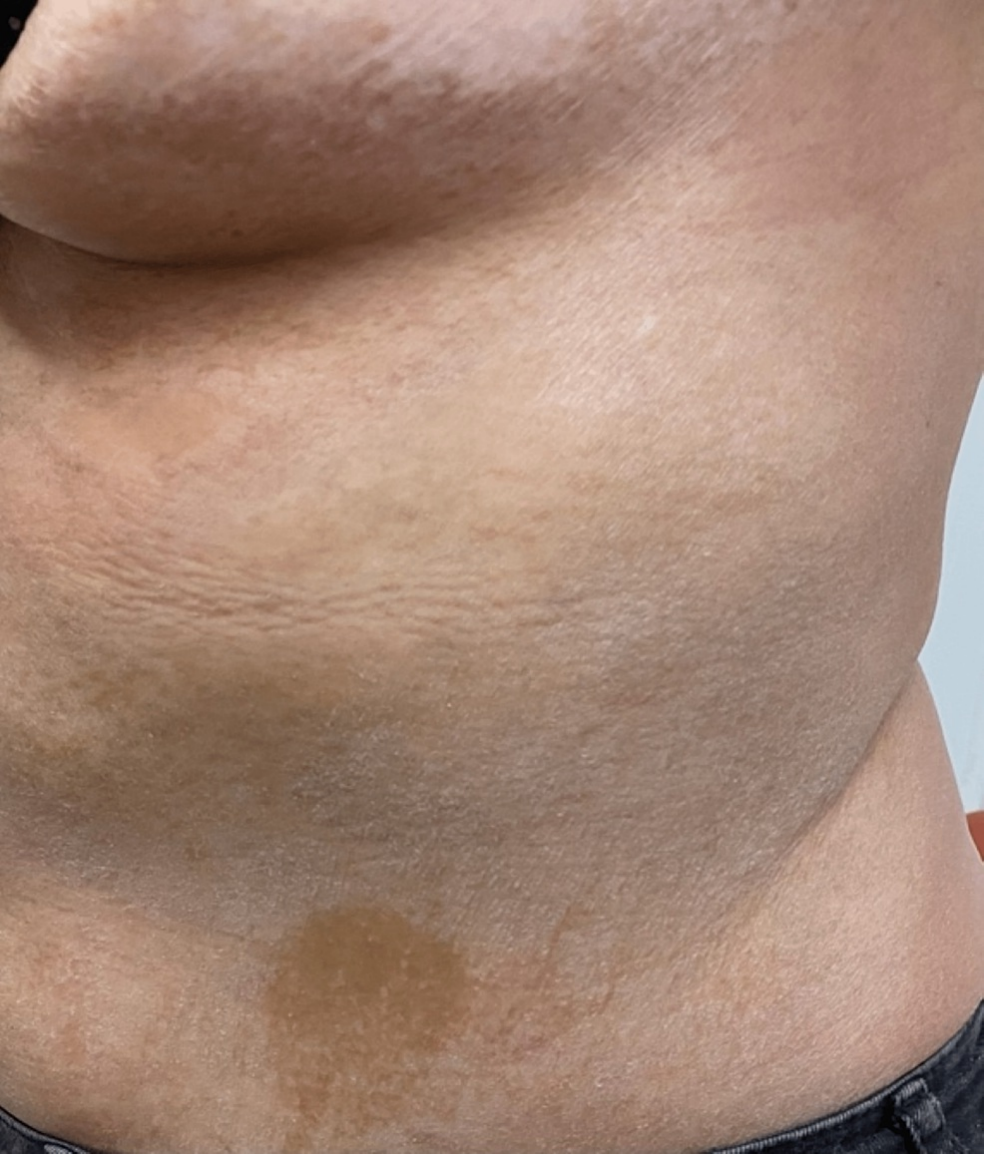
Hyperpigmented-to-silvery, indurated, sclerotic plaques

**Figure 2:**
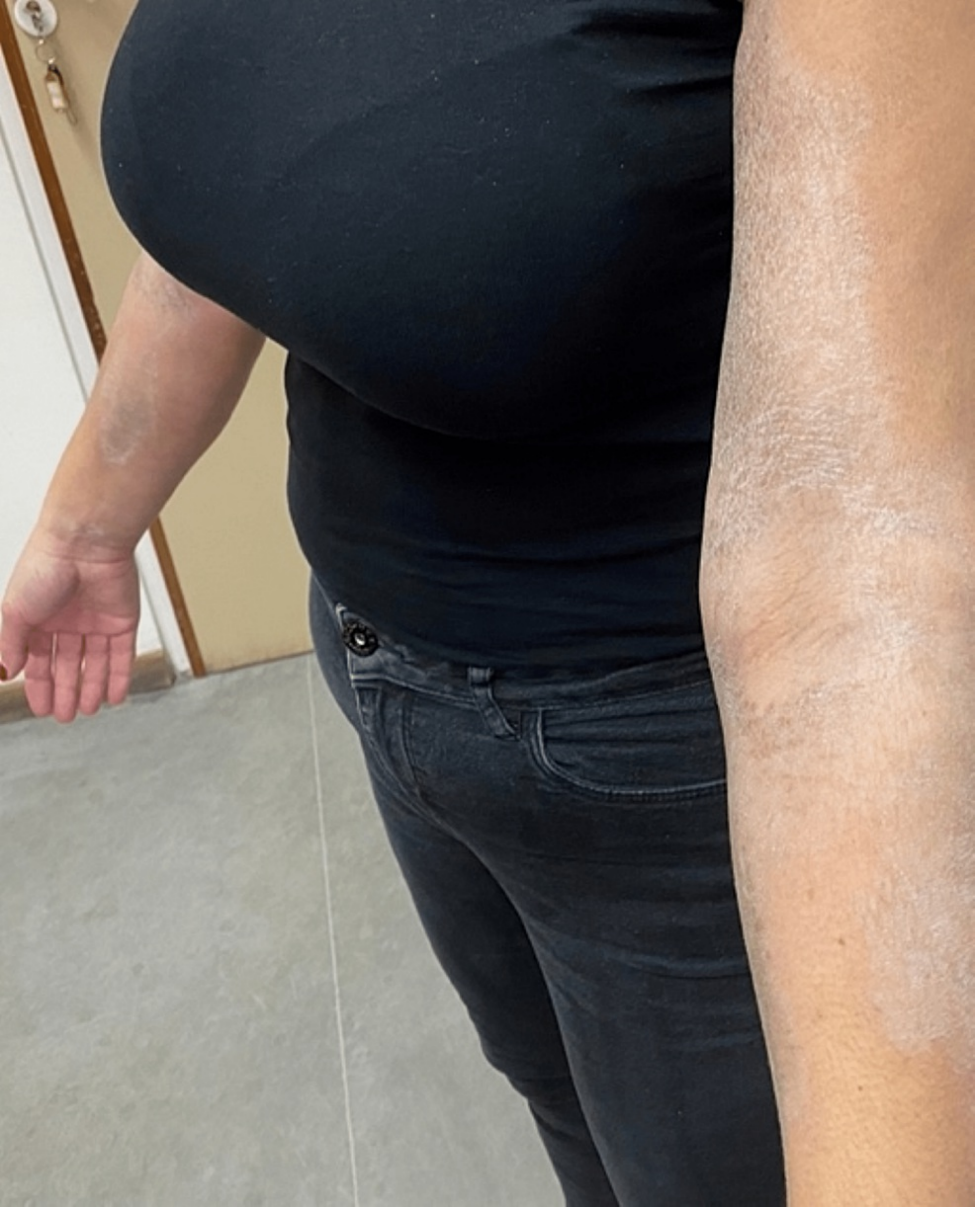
Hyperpigmented-to-silvery, indurated, sclerotic plaques

**Figure 3:**
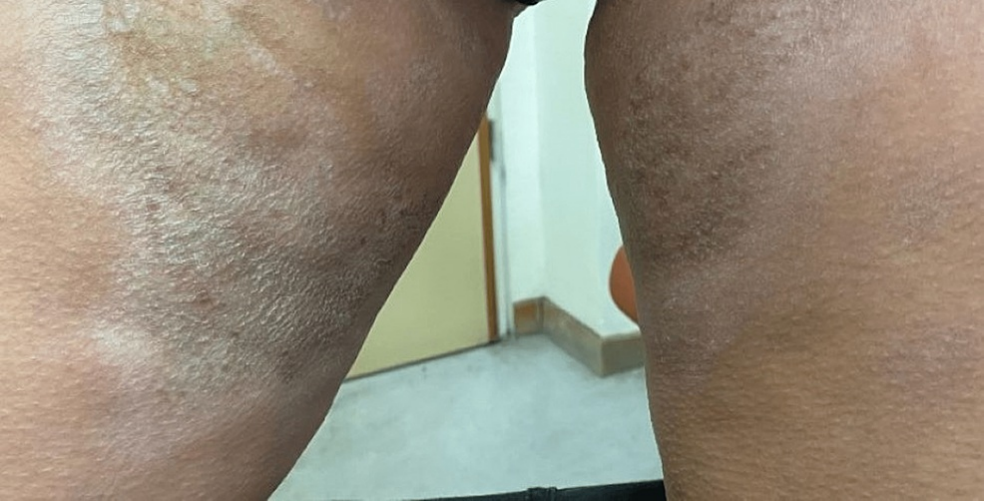
Hyperpigmented-to-silvery, indurated, sclerotic plaques

**Figure 4:**
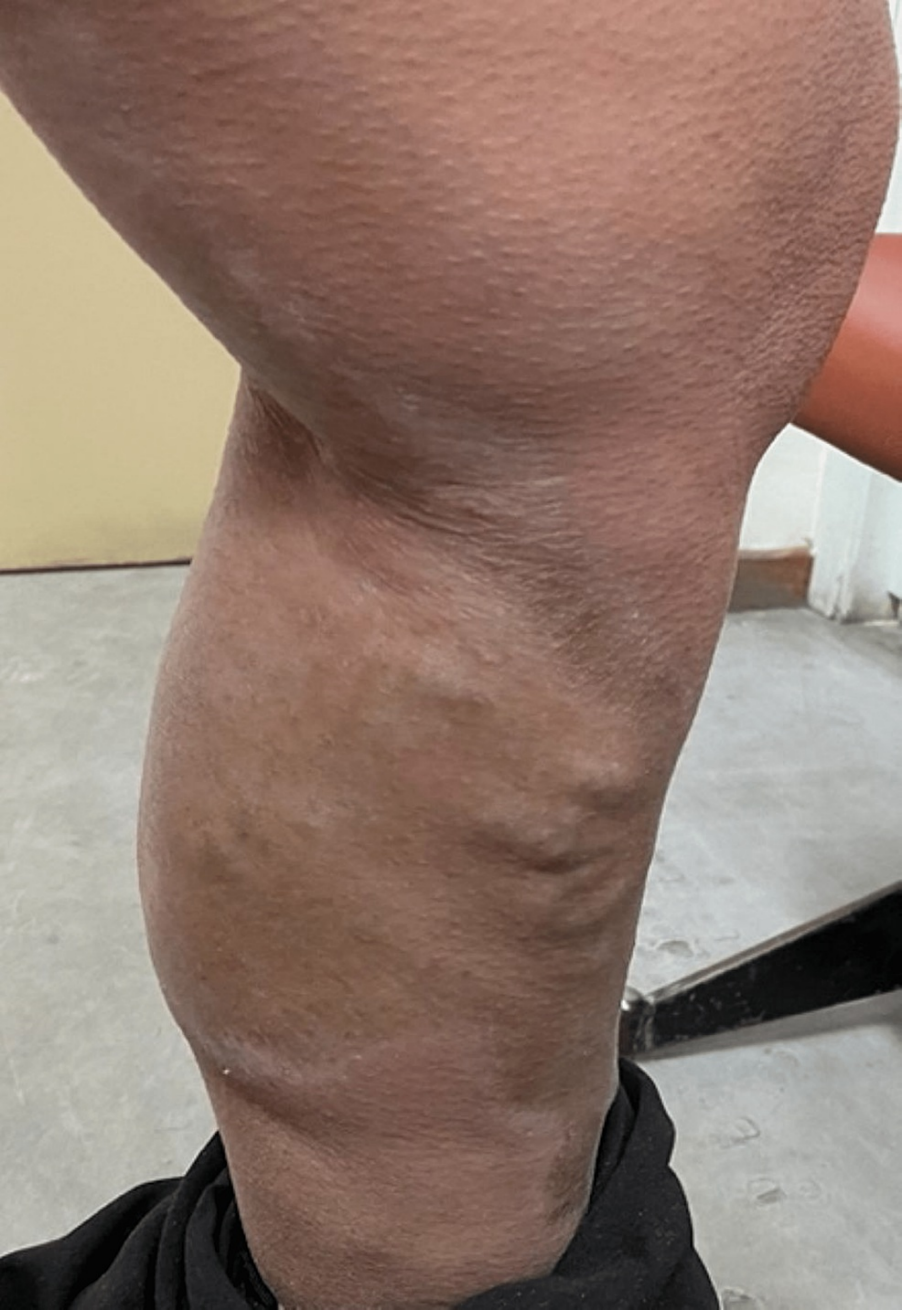
Hyperpigmented-to-silvery, indurated, sclerotic plaques

Dermatological examination revealed the presence of multiple hyperpigmented-to-silvery, indurated, sclerotic plaques affecting the trunk, mainly the chest and upper back, as well as the extremities. The characteristic "salt-and-pepper" sign with hyperpigmentation areas alternating with hypopigmentation areas was also observed (Figures 1-4).

The lesions on the extremities showed a linear distribution along the lines of Blaschko. There were no nailfold capillary changes, sclerodactyly, Raynaud phenomenon, or findings of systemic involvement. The patient was a Fitzpatrick skin phototype IV.

A 4-mm punch biopsy was performed under the right breast, and the histopathological examination of the specimen revealed epidermal atrophy, focal hyperkeratosis, follicular plugging, and vacuolar interface damage. In addition, there was homogenization and edema of the papillary dermis. The deep dermis showed thickened collagen and perivascular lymphoplasmacytic cell infiltrate, mainly from lymphocytes and fewer histiocytes. These histopathological features were compatible with a mixed diagnosis of both LSA and morphea at the same lesion.

Laboratory testing for antinuclear antibodies (ANA), anti-Scl-70, anti-ds-DNA, and rheumatoid factor tests were negative.

The clinical and histopathology findings were consistent with the overlap of Lichen sclerosus et atrophicus and morphea. Systemic therapy with oral hydroxychloroquine 200mg twice daily was initiated. However, due to a reported photosensitivity rash, it was discontinued after one month of therapy. The patient was administered azathioprine orally (100 mg/daily), with a subsequent clinical improvement of the sclerotic plaques.

## Discussion

Lichen sclerosus et atrophicus (LSA) and morphea are two inflammatory dermatoses exhibiting considerable clinical and histopathological similarities [3]. Differentiating them can be a challenging clinical scenario as they tend to present similarly with white, sclerotic, and indurated plaques [1, 2, 5]. However, LSA patients experience intense pruritus more frequently than morphea patients [1]. Nonetheless, a few patients present with both conditions simultaneously on the same lesions [5]. These are more often women with widespread lesions of morphea. Additionally, the "salt-and-pepper" sign, characterized by a vitiligo-like depigmentation with perifollicular pigmentary retention, is considered one of the early signs of systemic sclerosis [6] and is most commonly found in patients with darker phototypes, such as our patient. However, our patient showed no clinical or laboratory evidence of systemic sclerosis.

The coexistence of LSA and morphea is well established [3, 5], but its etiology and pathogenesis still remain unknown. A retrospective study of 472 patients contacted by Kreuteur et al. showed the coexistence of LSA and morphea on histopathologic examination in 5.7% of morphea patients [5]. Similarly, there are a few case reports reporting LSA and morphea coexistence. Rongioletti et al. [7], Abhijit et al. [8], Almuqati et al. [6], and Rose et al. [9] report cases of histopathological coexistence of LSA and morphea but with no clear pathophysiologic connections or risk factors for both diseases. Furthermore, since the two entities have overlapping clinical and histopathological features, some argue that LSA is a subepidermal form of morphea [7]. On the other hand, others have postulated that there is a common triggering factor involving autoimmune pathways for both dermatoses [8]. It is, therefore, highly probable that these two inflammatory dermatoses belong to the same disease spectrum [5].

Until now, no clear genetic connection has been identified, despite their connection to the HLA-DR region [1.2]. On the contrary, epigenetic mechanisms and oxidative DNA damage may explain the coexistence of LSA and morphea. Small endogenous noncoding miR-155 plays an important role in LSA pathogenesis by enhancing Th1 immune response and regulating fibrotic tissue formation [1]. Wolska-Gawron et al. suggested that miR-155 acts as a fibrosis regulator in morphea too [10]. In addition, miRNA-155 expression seems to be upregulated in both diseases, leading to skin fibrosis, by regulating the Akt and Wnt/β-catenin pathways [1,10]. Galectin-7 seems to stimulate dermal fibroblast activity in LSA and systemic sclerosis, but there are no available data about morphea [1,11]. Finally, oxidative stress plays an important role in both LSA and morphea pathogenesis [1,2]. Lipid peroxidation in keratinocytes, oxidative DNA damage, and low concentrations of antioxidants are responsible for inflammation maintenance in LSA and morphea [1, 2].

## Conclusions

We present this interesting case of the coexistence of LSA and morphea along with Blaschko lines, aiming to draw attention to this uncommon phenomenon, which may indicate a possible etiological link between LSA and morphea. Morphea and LSA are considered to be two distinct immune-mediated diseases, presenting as dermal sclerosis and fibrosis. They share many similar clinical and histopathological features and tend to co-occur more frequently than expected.
